# Supplementary material for: Phylogeny of Crataegus (Rosaceae) based on 257 nuclear loci and chloroplast genomes: evaluating the impact of hybridization
Source: PeerJ. 2021 Oct 26;9:e12418. doi: 10.7717/peerj.12418 (PMC8555502; doi:10.7717/peerj.12418)
Supplement: Supplemental Information 1 — Pairs of single-locus trees for 19 loci (Table S1) for which the diploids-only trees (on the left; Dtreennn) had the highest values produced by the R treeConcordance function (treespace package; (Jombart et al., 2017; Kendall, 2019) calculated using the plastome reference tree collapsed by Crataegus taxonomic sections (Fig. 2C). On the right, trees for the diploid + tetraploid accessions (Treennn). Concordance values calculated using the corresponding plastome tree (Fig. 2D) similarly collapsed by Crataegus taxonomic sections. Individual accessions are numbered (snn) as in Table 1, and color-coded by Crataegus subgenus (diploid trees) and Crataegus section (diploid + tetraploid trees; see Table 1 for names). All trees were rooted using the corresponding sequences from the genome of apple, Malus ×domestica (Velasco et al., 2010); branch lengths were removed. [file peerj-09-12418-s001.pdf]

Dtree179

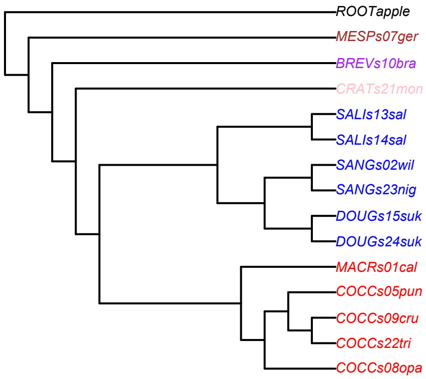

concordance = 0.75

Tree179

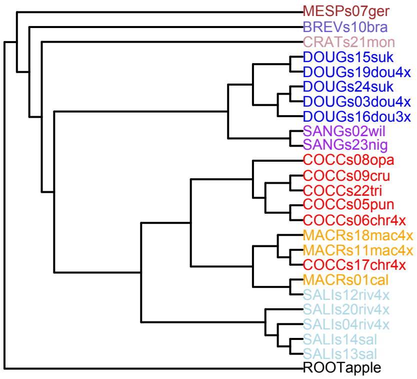

concordance = 0.418

Dtree5

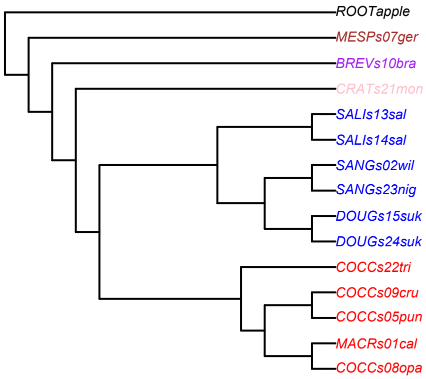

concordance = 0.719

Tree5

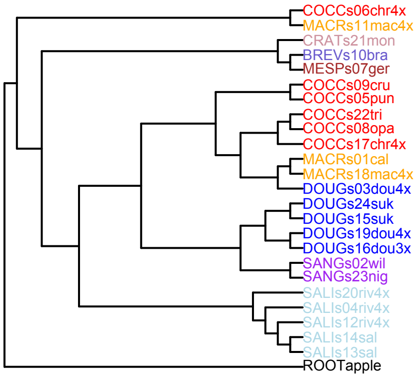

concordance = 0.349

Dtree254

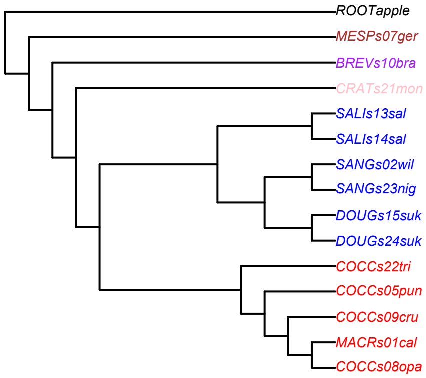

concordance = 0.719

Tree254

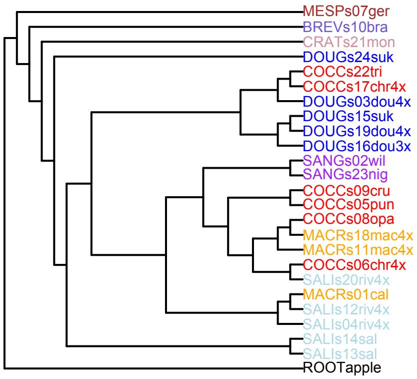

concordance = 0.287

Dtree198

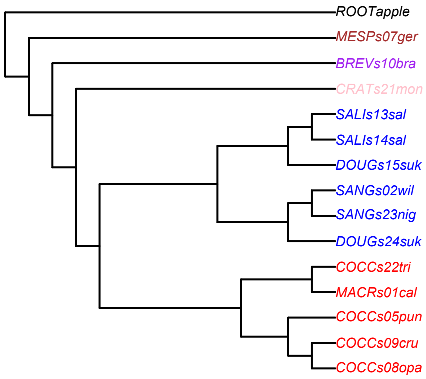

concordance = 0.698

Tree198

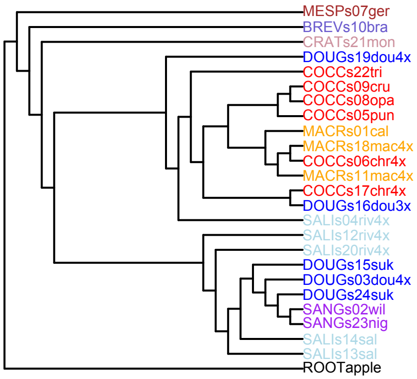

concordance = 0.51

Dtree233

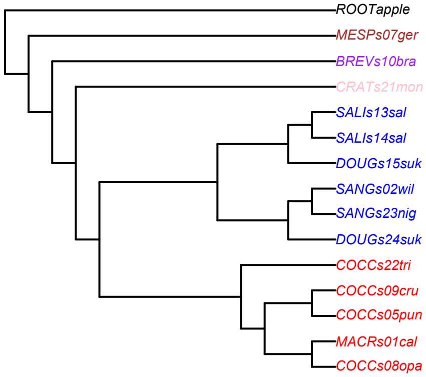

concordance = 0.677

Tree233

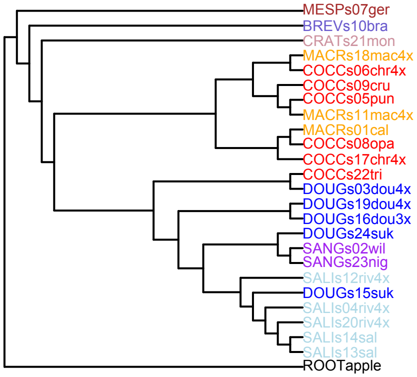

concordance = 0.605

Dtree17

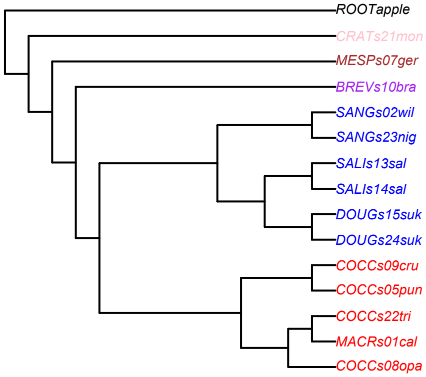

concordance = 0.667

Tree17

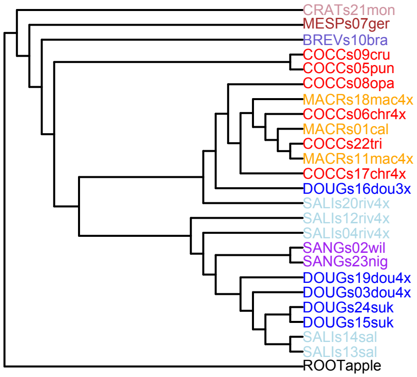

concordance = 0.314

Dtree121

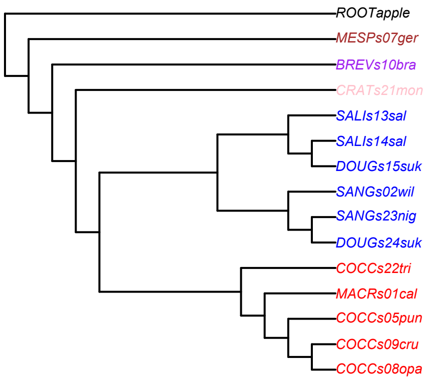

concordance = 0.667

Tree121

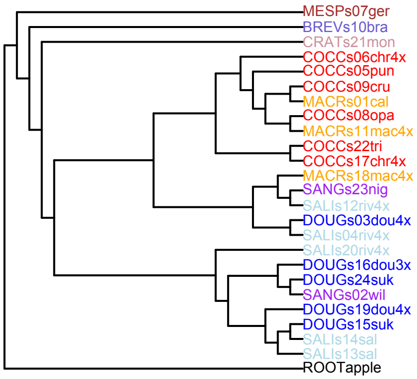

concordance = 0.506

Dtree127

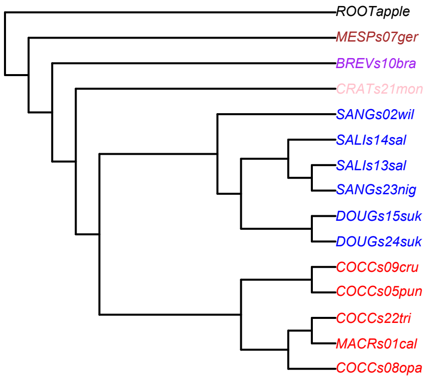

concordance = 0.646

Tree127

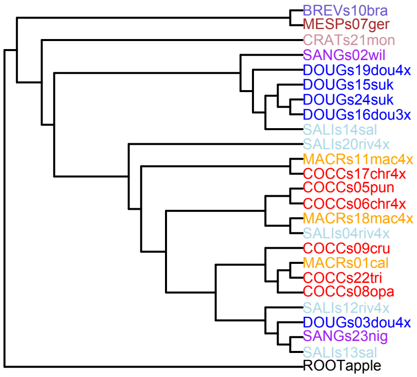

concordance = 0.169

Dtree63

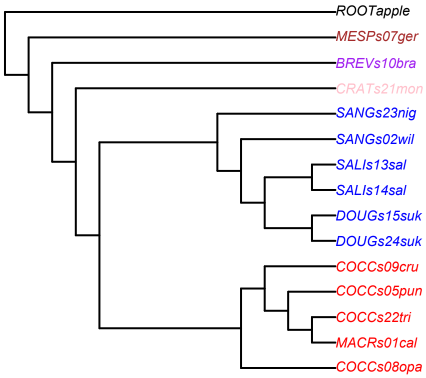

concordance = 0.635

Tree63

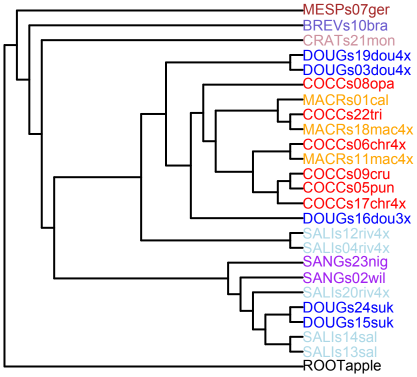

concordance = 0.46

Dtree90

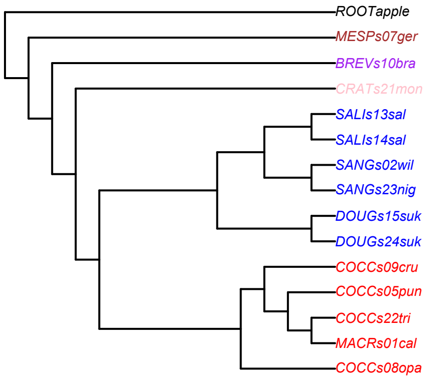

concordance = 0.635

Tree90

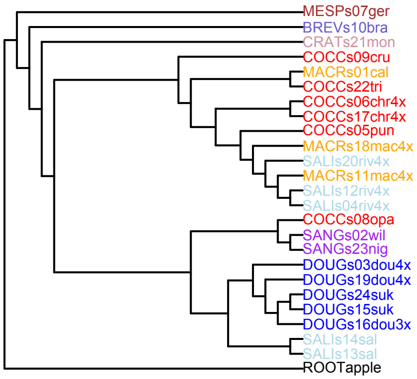

concordance = 0.49

Dtree95

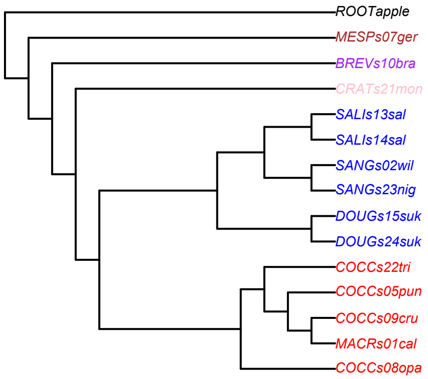

concordance = 0.635

Tree95

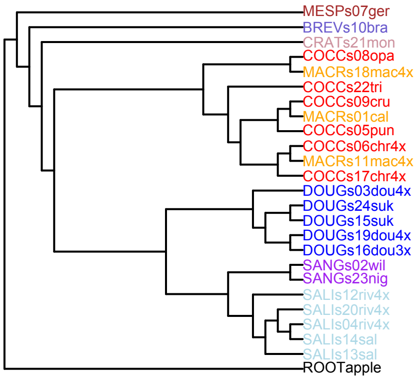

concordance = 0.713

Dtree129

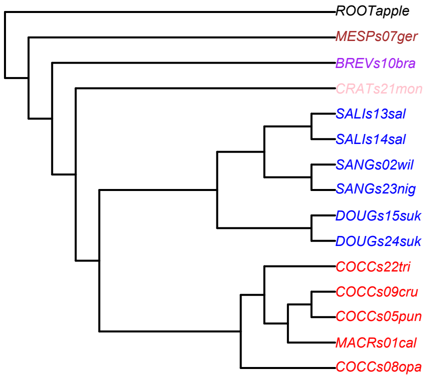

concordance = 0.635

Tree129

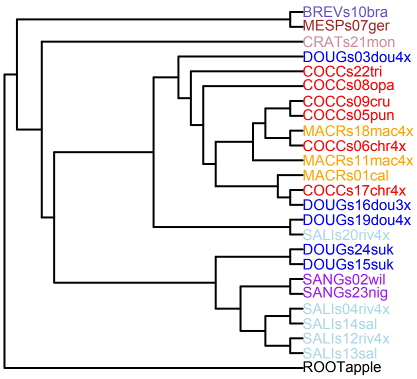

concordance = 0.207

Dtree232

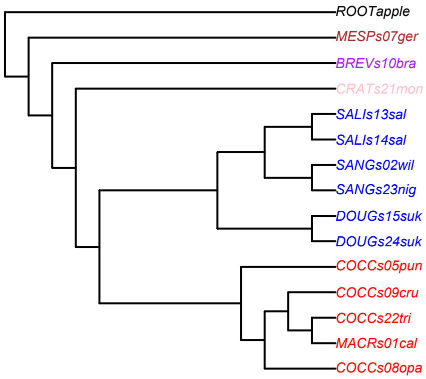

concordance = 0.635

Tree232

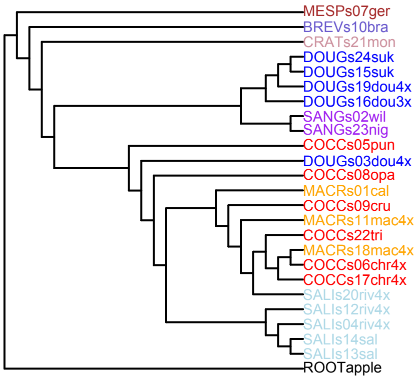

concordance = 0.395

Dtree237

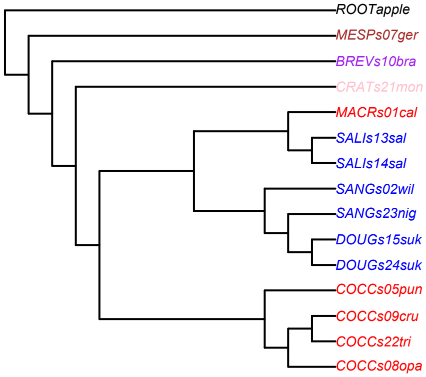

concordance = 0.625

Tree237

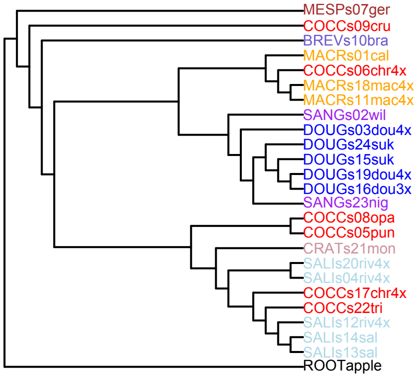

concordance = 0.303

Dtree22

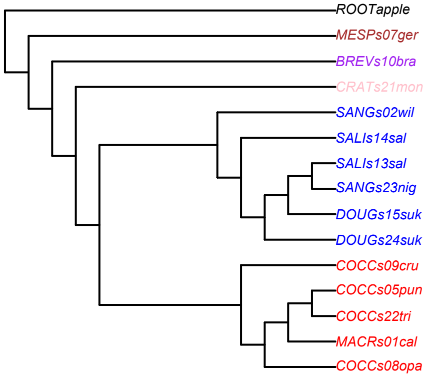

concordance = 0.615

Tree22

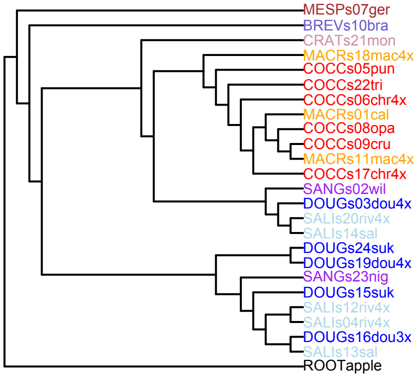

concordance = 0.192

Dtree58

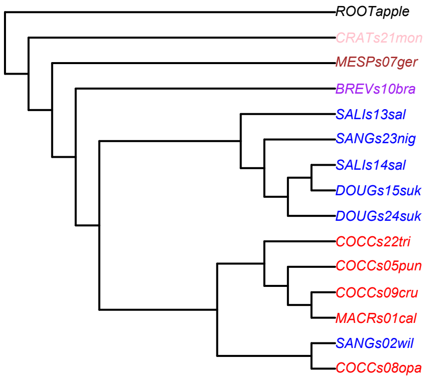

concordance = 0.615

Tree58

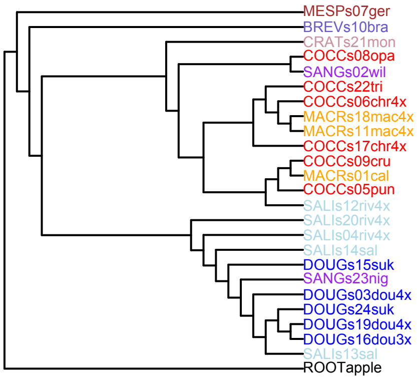

concordance = 0.215

Dtree222

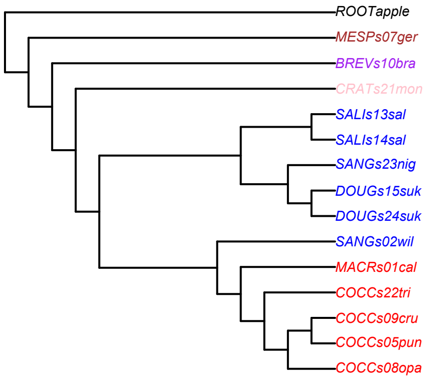

concordance = 0.615

Tree222

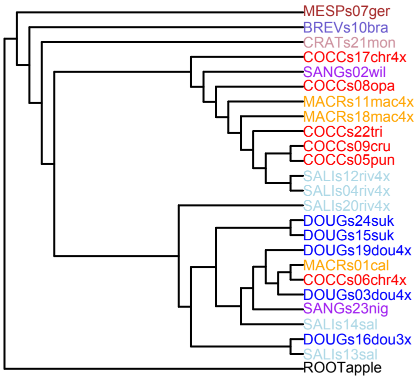

concordance = 0.475

Dtree91

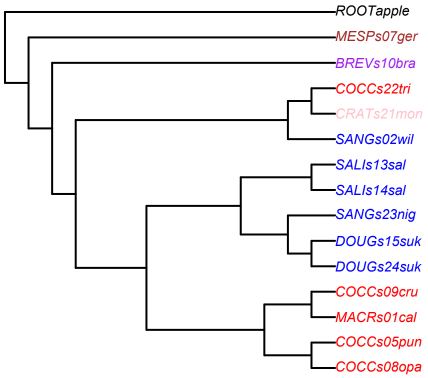

concordance = 0.594

Tree91

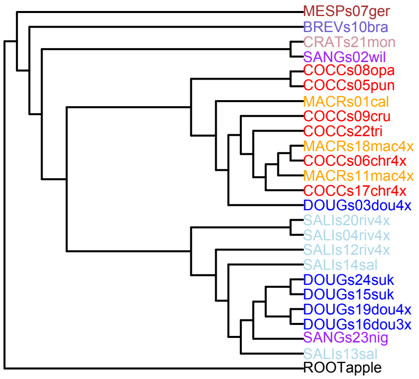

concordance = 0.582

Dtree208

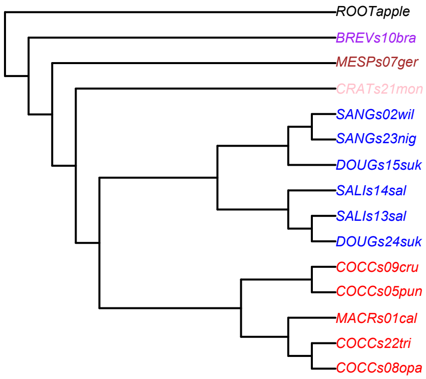

concordance = 0.573

Tree208

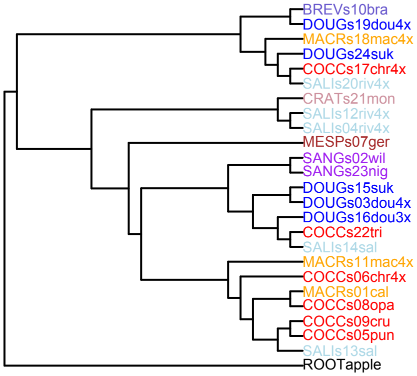

concordance = 0.352
