# Supplementary figures and images for: Phylogeny of Crataegus (Rosaceae) based on 257 nuclear loci and chloroplast genomes: evaluating the impact of hybridization
Source: PeerJ. 2021 Oct 26;9:e12418. doi: 10.7717/peerj.12418 (PMC8555502; doi:10.7717/peerj.12418)

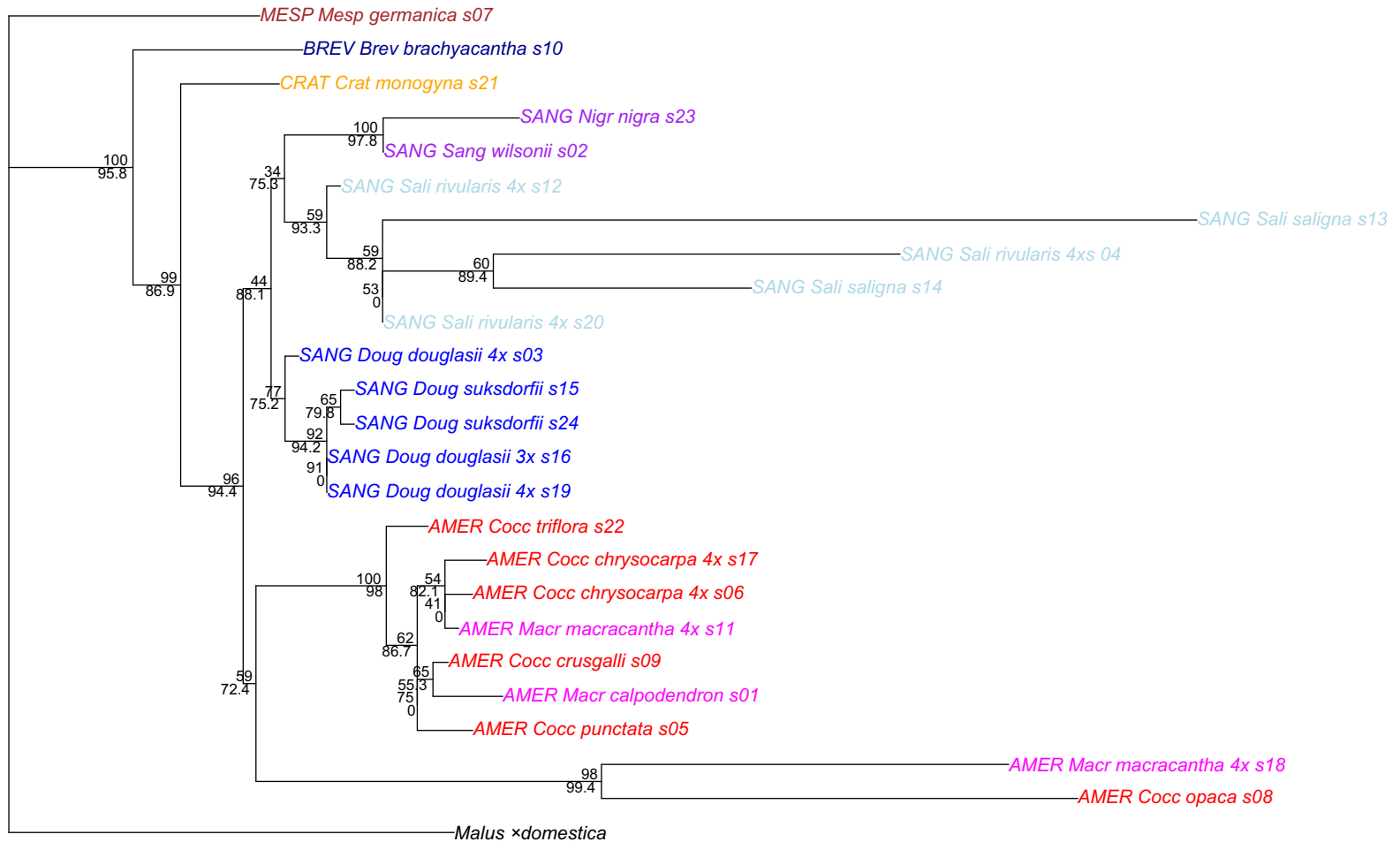

Supplement: Supplemental Information 2 — Phylogenetic tree for the diploids+polyploids sample and locus 95 (MDP0000220167, on chromosome 8, probable Vacuolar proton translocating ATPase 100 kDa subunit; Table S1). Branch support given by %ultrafast bootstrap support (above; (Hoang et al., 2018)) and %SH-aLRT support (below; (Guindon et al., 2010)). Tree rooted using the corresponding sequence from the genome of apple, Malus ×domestica (Velasco et al., 2010). Scale bar for branch lengths is in substitution units. [file peerj-09-12418-s002.pdf]

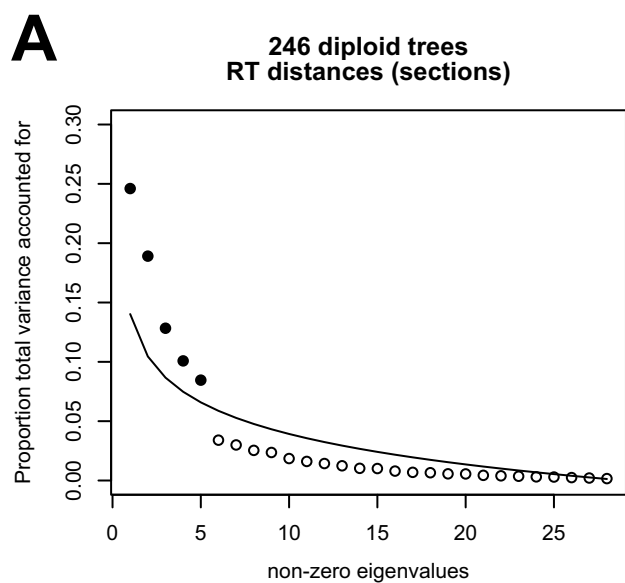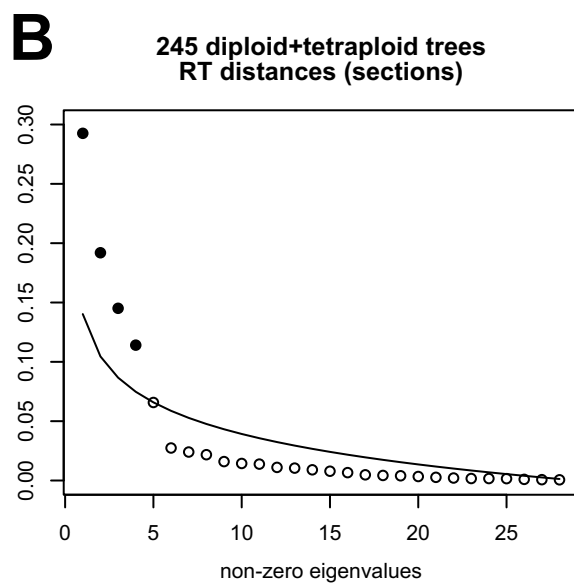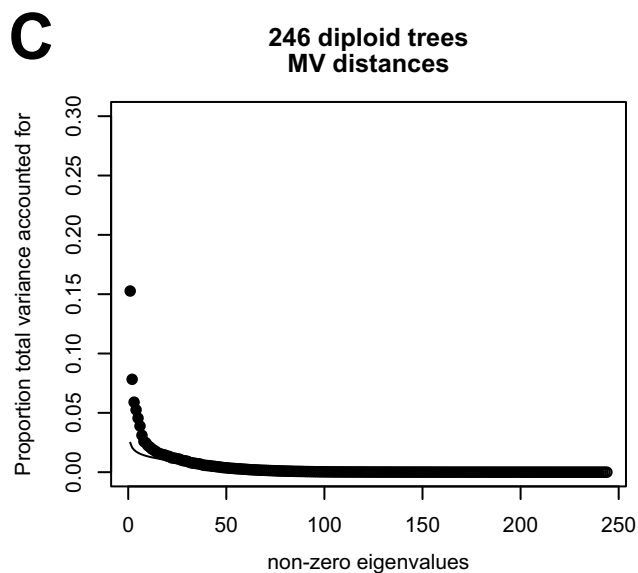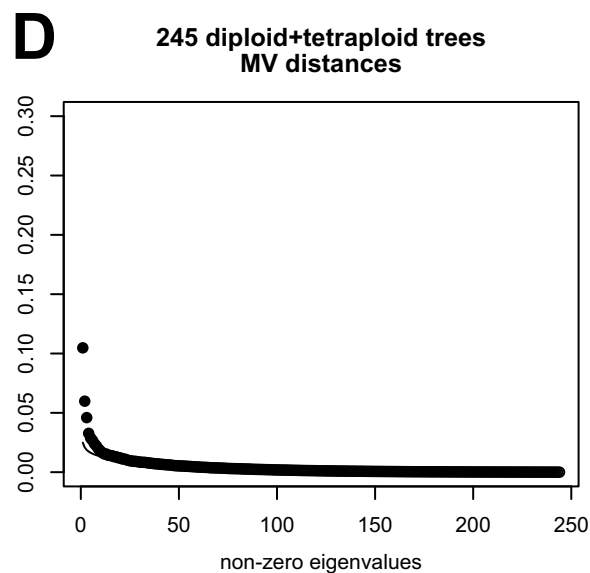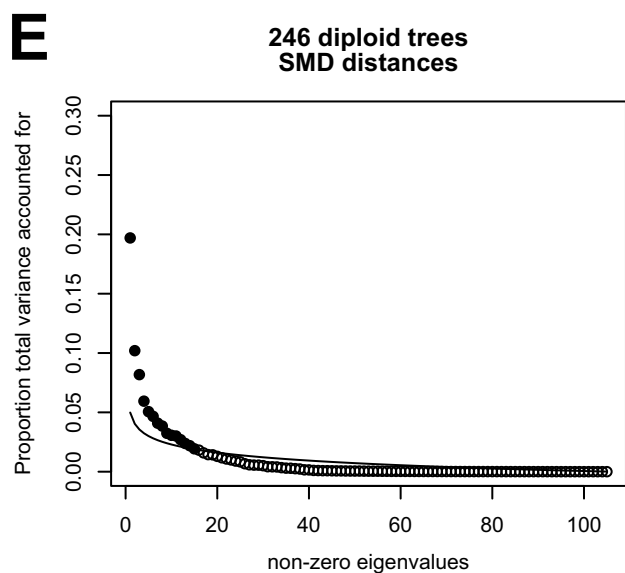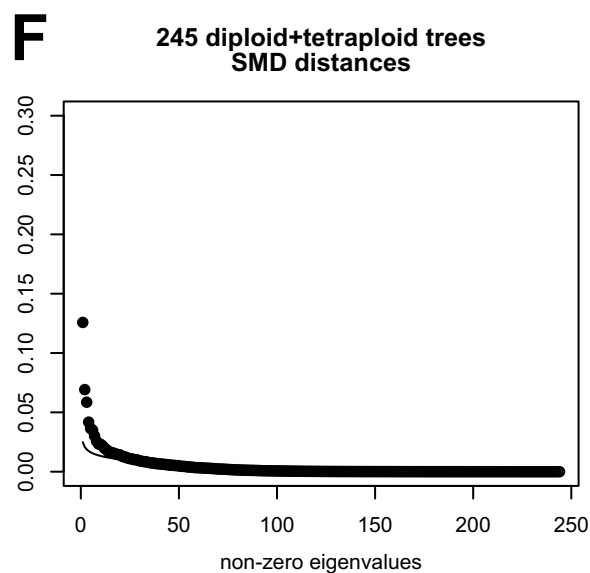

Supplement: Supplemental Information 4 — Proportion of the total variance accounted for plotted for the non-zero eigenvalues of the transformed distance matrices, arranged from largest to smallest. Also plotted is the line connecting the corresponding proportions expected under the broken-stick model (Frontier, 1976; Legendre & Legendre, 1998); points for eigenvalues greater than these amounts are filled. Plots A-F correspond to the principal coordinates analyses in Figs. 4A–4F, respectively. (A, C, E) Eigenvalues of transformed distance matrices calculated for the diploid accessions only (plastome tree + 245 gene trees). (B, D, F) Eigenvalues of transformed distance matrices calculated for the diploid and tetraploid accessions (plastome tree + 244 gene trees). (A, B) Eigenvalues calculated from matrices of related tree (RT) distances between the trees. (C, D) Eigenvalues calculated from matrices of multivariate (MV) distances between the trees. (E, F) Eigenvalues calculated from matrices of subtree membership divergence (SMD) distances between the trees. [file peerj-09-12418-s004.pdf]
