# Supplementary material for: Phylogeny of Crataegus (Rosaceae) based on 257 nuclear loci and chloroplast genomes: evaluating the impact of hybridization
Source: PeerJ. 2021 Oct 26;9:e12418. doi: 10.7717/peerj.12418 (PMC8555502; doi:10.7717/peerj.12418)
Supplement: Supplemental Information 3 — Frequencies of estimated admixture ( [file peerj-09-12418-s003.pdf]

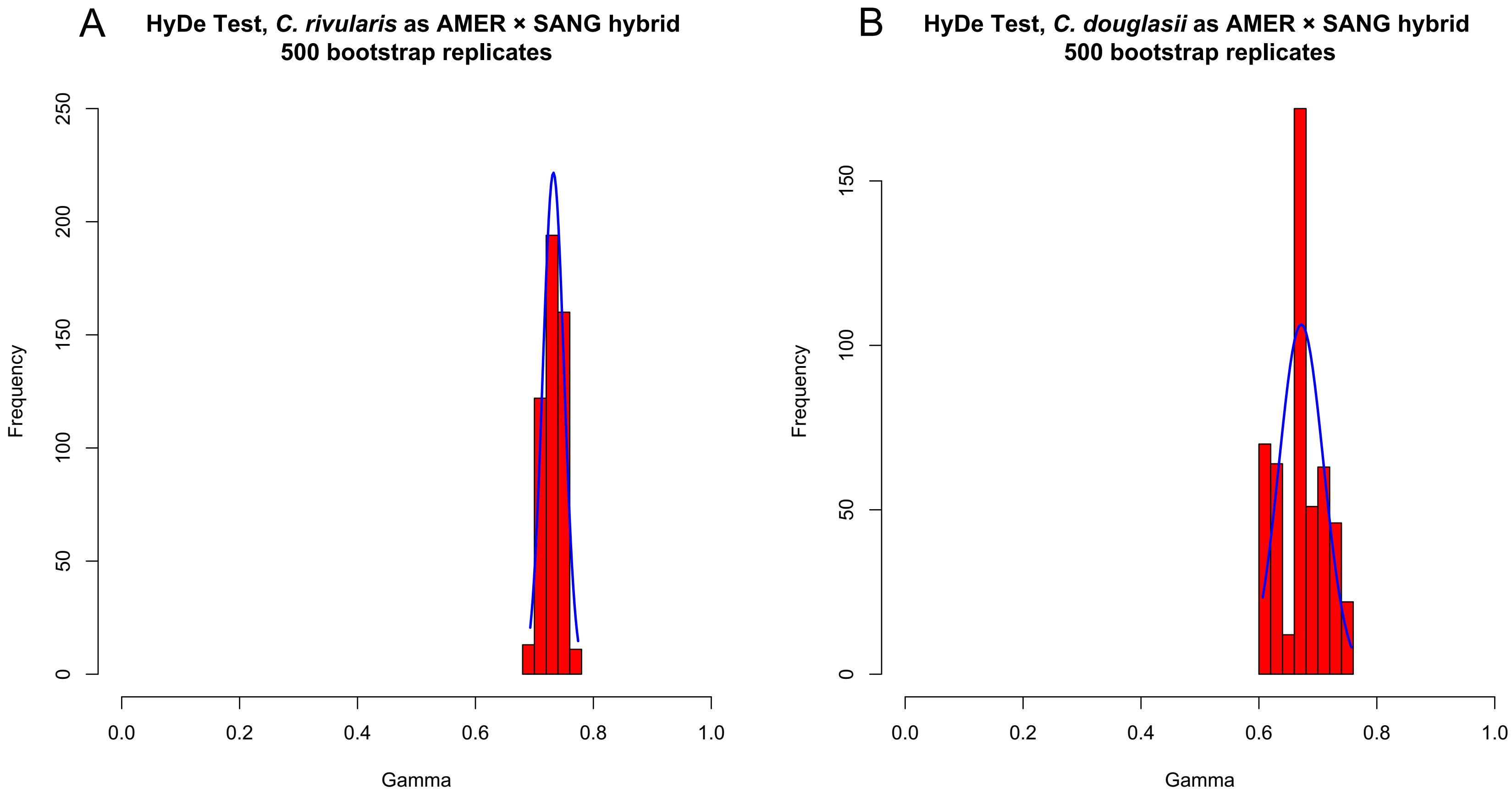
A
HyDe Test, *C. rivularis* as AMER × SANG hybrid
500 bootstrap replicates
Frequency
0
50
100
150
200
250
0.0
0.2
0.4
0.6
0.8
1.0
Gamma
B
HyDe Test, *C. douglasii* as AMER × SANG hybrid
500 bootstrap replicates
Frequency
0
50
100
150
0.0
0.2
0.4
0.6
0.8
1.0
Gamma
