# Supplementary material for: Phylogeny of Crataegus (Rosaceae) based on 257 nuclear loci and chloroplast genomes: evaluating the impact of hybridization
Source: PeerJ. 2021 Oct 26;9:e12418. doi: 10.7717/peerj.12418 (PMC8555502; doi:10.7717/peerj.12418)
Supplement: Supplemental Information 8 — Calculated by IQ-TREE (Minh, Hahn & Lanfear, 2020a) from concatenated gene sequences for 14 diploid Crataegus accessions (a; Fig. 5B; 245 loci) and for the same 14 diploids plus 10 related allotetraploid Crataegus accessions (b; Fig. 5C; 244 loci). ID, Branch ID (diploids only, Fig. 5B; diploids plus tetraploids, Fig. 5C); gCF, Gene concordance factor (%); gDF1, Gene discordance factor (%) for NNI-1 branch; gDF2, Gene discordance factor (%) for NNI-2 branch; gN, Number of trees decisive for the branch; sCF, Site concordance factor (%) averaged over 100 quartets; sDF1, Site discordance factor (%) for alternative quartet 1; sDF2, Site discordance factor (%) for alternative quartet 2; sN, Number of informative sites averaged over 100 quartets; Label, bootstrap support; Length, Branch length. [file peerj-09-12418-s008.docx]

**Supplementary Table S4:**

**Impact on gene and site concordances and discordances brought about by including allotetraploids in the multilocus ML phylogeny of diploid *Crataegus* accessions.** Calculated by IQ-TREE (Minh et al. 2020) from concatenated gene sequences for 14 diploid *Crataegus* accessions (a; Fig. 5b; 245 loci) and for the same 14 diploids plus 10 related allotetraploid *Crataegus* accessions (b; Fig. 5c; 244 loci). ID, Branch ID (diploids only, Fig. 5b; diploids plus tetraploids, Fig. 5c); gCF, Gene concordance factor; gDF1, Gene discordance factor for NNI-1 branch; gDF2, Gene discordance factor for NNI-2 branch; gN, Number of trees decisive for the branch; sCF, Site concordance factor averaged over 100 quartets; sDF1, Site discordance factor for alternative quartet 1; sDF2, Site discordance factor for alternative quartet 2; sN, Number of informative sites averaged over 100 quartets; bootstrap support.

1. **Diploids only sample**

ID %gCF %gDF1 %gDF2 gN %sCF %sDF1 %sDF2 sN %BS Branch length^[[1]](#footnote-1)^

16 41.22 15.92 15.51 245 46.25 23.93 29.82 2009.01 100 0.00298465

17 38.37 5.31 7.76 245 52.08 23.64 24.27 2012.75 100 0.00305184

18 25.71 3.27 4.08 245 45.67 28.66 25.67 1724.34 100 0.00195949

19 26.94 0 1.22 245 53.51 22.22 24.27 1781.22 100 0.00366421

20 6.94 6.94 8.57 245 33.78 34.01 32.21 1601.28 78 0.00107116

21 11.84 10.61 11.84 245 34.38 34.49 31.13 1536.61 100 0.0016004

22 17.55 4.9 6.53 245 41.8 27.15 31.05 1498.31 100 0.00198238

23 22.45 1.22 0.82 245 49.26 27.17 23.57 1738.87 100 0.00259658

24 10.61 7.76 5.71 245 33.11 34.49 32.4 1665.63 92 0.00110684

25 36.33 3.27 8.16 245 44.5 21.62 33.88 1830.36 100 0.00294373

26 62.45 2.04 0.41 245 65.75 18.08 16.17 2153.46 100 0.00935816

27 22.45 2.86 12.65 245 35.22 25.67 39.11 1707 99 0.00223505

**(b) Diploids plus Tetraploids**

26 40.98 15.57 14.75 244 45.64 24.19 30.18 1979.14 100 0.00295782

27 37.7 5.74 6.56 244 52.79 23.16 24.04 1954.22 100 0.00291973

28 26.23 0 0.41 244 50.85 24.91 24.24 1692.67 100 0.00202481

29 4.1 0 0.41 244 49.8 25.55 24.65 1672.58 100 0.00296349

30 5.33 2.46 1.23 244 35.49 31.99 32.52 1447.29 99 0.00196123

31 0.82 0.82 2.46 244 35.8 32.31 31.89 1533.65 69 0.000633141

32 1.23 0 1.23 244 34.78 32.05 33.17 1489.33 81 0.000775413

33 2.46 0 2.46 244 36.49 27.12 36.39 1398.33 98 0.00121097

34 13.93 6.97 3.69 244 39.35 29.64 31.01 1301.14 100 0.0014934

35 5.33 0.41 0.41 244 39.97 27.7 32.33 1309.4 100 0.0012821

36 19.67 7.79 4.1 244 44.53 23.32 32.15 1254.77 100 0.00196944

37 2.46 0 0.41 244 40.85 26.65 32.5 1672.67 83 0.00146761

38 4.51 1.23 2.05 244 36.64 28.86 34.5 1626.72 75 0.0010574

39 7.79 0 0.82 244 36.52 23.64 39.84 1658 68 0.00137072

40 5.74 9.02 1.23 244 29.95 37.57 32.48 1725.65 64 0.00148941

41 16.8 5.33 4.92 244 48.61 30.65 20.75 1563.79 100 0.00245996

42 26.64 1.64 1.23 244 46 26.92 27.09 1684.16 100 0.00233574

43 24.59 0 2.46 244 43.77 25.39 30.84 1634.54 100 0.00258685

44 12.7 0 0 244 44.5 22.9 32.6 1794.62 100 0.00314622

45 8.2 7.79 9.43 244 39.26 32.94 27.8 1746.79 100 0.00239818

46 9.02 9.84 14.34 244 40.26 33.59 26.15 1739.94 98 0.00213926

47 39.34 4.51 5.33 244 54.51 23.79 21.7 1911.77 100 0.00486515

Minh BQ, Hahn MW, and Lanfear R. 2020. New methods to calculate concordance factors for phylogenomic datasets. *Molecular Biology and Evolution*. 10.1093/molbev/msaa106

1. In substitution units [↑](#footnote-ref-1)
